# A New Method of RNA Secondary Structure Prediction Based on Convolutional Neural Network and Dynamic Programming

**DOI:** 10.3389/fgene.2019.00467

**Published:** 2019-05-22

**Authors:** Hao Zhang, Chunhe Zhang, Zhi Li, Cong Li, Xu Wei, Borui Zhang, Yuanning Liu

**Affiliations:** ^1^College of Computer Science and Technology and Symbol Computation and Knowledge Engineering, Ministry of Education, Jilin University, Changchun, China; ^2^College of Computer Science and Technology, Changchun University of Science and Technology, Changchun, China; ^3^Columbia Independent School, Columbia, MO, United States

**Keywords:** convolutional neural network, dynamic programming, RNA secondary structure, base pairing probability, energy balance status

## Abstract

In recent years, obtaining RNA secondary structure information has played an important role in RNA and gene function research. Although some RNA secondary structures can be gained experimentally, in most cases, efficient, and accurate computational methods are still needed to predict RNA secondary structure. Current RNA secondary structure prediction methods are mainly based on the minimum free energy algorithm, which finds the optimal folding state of RNA *in vivo* using an iterative method to meet the minimum energy or other constraints. However, due to the complexity of biotic environment, a true RNA structure always keeps the balance of biological potential energy status, rather than the optimal folding status that meets the minimum energy. For short sequence RNA its equilibrium energy status for the RNA folding organism is close to the minimum free energy status; therefore, the minimum free energy algorithm for predicting RNA secondary structure has higher accuracy. Nevertheless, in a longer sequence RNA, constant folding causes its biopotential energy balance to deviate far from the minimum free energy status. This deviation is because of its complex structure and results in a serious decline in the prediction accuracy of its secondary structure. In this paper, we propose a novel RNA secondary structure prediction algorithm using a convolutional neural network model combined with a dynamic programming method to improve the accuracy with large-scale RNA sequence and structure data. We analyze current experimental RNA sequences and structure data to construct a deep convolutional network model, and then we extract implicit features of an effective classification from large-scale data to predict the pairing probability of each base in an RNA sequence. For the obtained probabilities of RNA sequence base pairing, an enhanced dynamic programming method is applied to obtain the optimal RNA secondary structure. Results indicate that our proposed method is superior to the common RNA secondary structure prediction algorithms in predicting three benchmark RNA families. Based on the characteristics of deep learning algorithm, it can be inferred that the method proposed in this paper has a 30% higher prediction success rate when compared with other algorithms, which will be needed as the amount of real RNA structure data increases in the future.

## Introduction

RNA is an important basic substance in living organisms. It plays an important role in encoding, decoding, regulating, and expressing genes. The function of RNA in an organism depends mainly on its tertiary structure. However, the tertiary structure of RNA molecules is complex and lacks an effective representation to describe it; thus, it is very difficult to directly predict the tertiary structure from the primary structure of RNA molecules. Therefore, predicting the secondary structure of RNA from the primary structure of RNA becomes the main process for studying RNA structure.

At present, the identified RNA secondary structure can be obtained mainly by means of biological experiments such as X-ray diffraction and NMR. However, biological experimental methods are inefficient, expensive, and arduous when measuring structures on large scales (Novikova et al., 2012); furthermore, they are not effective for all RNA molecules (Fürtig et al., 2003). Howard and Eran proposed the PARS technique to predict the RNA secondary structure (Kertesz et al., 2010). It applies endonucleases to cleave the single-stranded portion and the double-stranded portion of the RNA to create a library of two RNA fragments, and then sequence-analyzes the two RNA fragment libraries separately to obtain an RNA secondary structure. But endonucleases cannot pass through the cell membrane, and RNA can only be extracted from the cells. This will destroy an RNA natural structure and result in structural changes. Ding et al. (2014) uses DMS for biological experiments. DMS can react with adenine and cytosine in unpaired RNA sequences in cells, and RNA regions reactive with DMS cannot be reverse transcribed into DNA. The DNA reverse-transcribed into RNA is subjected to sequence analysis to determine unpaired RNA regions. DMS technology still has drawbacks. It can only determine two paired nucleotides in an RNA molecule, and the rest requires computer algorithms for simulation. In addition, researchers have used SHAPE reagents instead of DMS reagents (Wilkinson et al., 2008; Novikova et al., 2013), which can acylate the 2' hydroxyl groups of four bases in an unpaired state, thereby analyzing the single-strand flexibility of the RNA backbone at any position and speculating whether the bases are paired. However, the pairing object cannot be determined. Up until now, not one biological RNA method has been able to predict a true RNA secondary structure in large quantities; thus, computational prediction algorithms are still needed to effectively predict RNA secondary structures.

There are two main types of mainstream RNA secondary structure prediction algorithms. One is the deterministic dynamic programming algorithm. The earliest use of a dynamic programming algorithm is the Nussinov algorithm based on the maximum number of base pairings (Nussinov et al., 1978). This algorithm simply assumes that the RNA single-strands are folded into themselves so that base pairs can (as much as possible) constitute the secondary structure of the RNA. However, this algorithm has low prediction accuracy due to the assumption that the premise is too simple, and the formed base pairs are often discontinuous and cannot form stem regions. Based on the Nussinov algorithm and energy information, Zuker proposed a minimum free energy algorithm (Zuker and Stiegler, 1981). The minimum free energy algorithm assumes that RNA structure has a great relationship with free energy. The size of free energy is not only related to the type of base pairing, but the free energy size is also affected by adjacent base pairs. The free energy of different structures (hair-loop, inner-loop, etc.) is also very different. The minimum free algorithm still uses the idea of dynamic programming, but the calculated object is a series of complex free energy parameters obtained from experiments. Many well-known RNA secondary structure prediction software applications, such as the mfold web server (Zuker, 2003) and RNAfold (Hofacker et al., 1994), have adopted the minimum free algorithm and its improvement. However, experiments show that due to the complexity of the internal environment, RNA is seldom folded in a manner that can minimize the free energy of the structure, and it is generally in a suboptimal energy folded structure (Zou et al., 2008). Notably, the Zuker algorithm has better prediction results for secondary structures of shorter RNAs. However, for longer RNAs, its prediction accuracy acutely decreases.

The second category of mainstream RNA secondary structure prediction algorithms refers to the comparative sequence analysis methods. In biological experiments, it is usually necessary to simultaneously process one or more sets of homologous RNA sequences. It is generally believed that in homologous RNA molecules, the conservation of the structure is greater than the conservation of the sequence. For example, the secondary structures of all tRNA molecules are clover-shaped. This consistency of shape gives tRNA molecules the structural consistency they need to perform similar functions. Therefore, the comparing sequence method can improve prediction accuracy to a certain extent. There are three main methods of comparative sequence analysis. The first method includes a prior distribution of RNA structures, which includes evolutionary history when comparing and post-predicting (Knudsen and Hein, 1999). The results obtain by this method strongly depend on the effect of multiple sequence alignment. The second method simultaneously performs structural prediction and sequence comparison, but this algorithm consumes excessive computational resources (Sankoff, 1985). The third comparative sequence analysis method predicts first and compares afterwards. This method can obtain multiple candidate structures, but it cannot be guaranteed to contain real structures (Allali and Sagot, 2005).

Artificial intelligence methods have been applied in many fields. At present, there have been some artificial intelligence learning algorithms such as the genetic algorithm (Hu, 2003), neural network algorithm (Zhang et al., 2006), support vector machine algorithm, and other methods to predict the secondary structure of RNA. All achieved good results. However, all these methods are based on small samples, and the prediction accuracy is low for single-class data samples. With the development of computer technology, deep learning methods have emerged in the field of artificial intelligence, which can effectively improve the accuracy of prediction. Deep learning methods can extract effective and implicit features through deep-seated networks in large-scale data and use these features to construct effective prediction models. At present, deep learning methods have made great breakthroughs in the field of protein secondary structure prediction (Wang et al., 2016). However, compared with secondary structure prediction of proteins, RNA secondary structure prediction is more complicated and difficult since each pair of bases on the RNA needs to correspond to another base in the chain even though each amino acid of a protein is not related to other amino acids in the chain during structure prediction. This paper proposes a novel computational method that combines deep learning with dynamic programming to predict RNA secondary structure prediction, which can effectively solve the problems above. Compared with the current mainstream algorithms, our method has better results.

## Data and Methods

**Figure 1:**
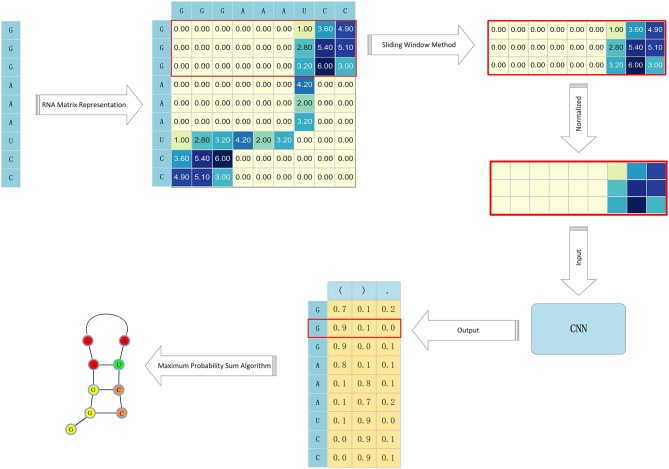
The process of CDPfold.

The RNA secondary structure is mainly composed of a stem structure formed by complementary pairing of contiguous bases and a cyclic structure formed by non-pairing of bases. This RNA secondary structure is also called the stem-and-loop structure, As long as all the paired bases of an RNA sequence are determined, the secondary structure of the entire RNA can be determined. Based on the RNA secondary prediction problems presented in our literature search up to this point, this paper proposes a more efficient algorithm for RNA secondary structure prediction. This algorithm, referred to as CDPfold, combines a convolutional neural network and dynamic programming as well as a sequence alignment method. In comparative sequence analysis, we constructed a convolutional neural network to extract the characteristics of effective implicit features from large-scale data and predicted the matching probability of each base on the RNA sequence. Convolutional neural networks can use the currently collected RNA sequences as training samples, which solves the constraints of homologous sequences in comparative sequence analysis. For the probabilistic results obtained by the convolutional neural network, we used the iterative idea of dynamic programming and the definition of the RNA secondary structure to obtain the base matching probability and the maximum RNA secondary structure. This operation can avoid the degradation of long sequence prediction accuracy due to the use of the free energy method. The process of CDPfold predicting an RNA secondary structure is shown in Figure 1.

### RNA Matrix Representation Based on RNA Sequence Pairing

An RNA sequence is mainly composed of four types of base combinations, “A,” “U,” “G,” and “C,” but most of the algorithm models do not accept the “AUGC” combination sequence as input data. So, we had to encode the sequence. Currently, the most common encoding method is one-hot encoding, but since one-hot encoding does not reflect the implicit matching between bases, we developed a new encoding method.

We built the matrix *W*_*i*×*i*_ for each RNA, where each row of the matrix represents possible pairings of bases at that position, as follows:

1. According to the number of hydrogen bonds between the paired bases, the pairing weight between A and U is set to 2, and the pairing weight between G and C is set to 3. Since the U-G pair is a wobble base pair, the pairing weight between U and G is set to *x* (0 < *x* < 2), which leads to:
  (1) $$P(R_{j},R_{j})=\;\{\begin{array}{ll} 2, & \;(if\;(R_{j}=A\;and\;Rj=\;U)\;or\;(R_{i}\;=\;U\;and\;R_{j}=A))\\ 3, & \left(if\;(R_{i}=\;G\;and\;R_{j}=\;C)\;or\;(R{\;}_{i}\;=\;C\;and\;R_{j}=G)\right)\\ x, & \left(if\;(R_{i}=\;G\;and\;R_{j}=\;U)\;or\;(R_{i}=\;U\;and\;R_{j}=G)\right)\\ 0, & else \end{array}$$
2. For any two positions, such as *i, j* on the RNA sequence, this article must not only consider the pairing of these two bases, but also whether these two positions can form paired bases on the stem. Therefore, we had to take into account the pairing of the bases on the left (right) side of *i* with the right (left) side of *j*.
3. For one stem, the paired bases in the middle of the stem are relatively stable, and the paired bases on both sides are relatively unstable. Therefore, the calculation offers the possibility of pairing the two positions, *i and j*, on an RNA sequence. This article refers to the idea of the local weighted linear regression, adding a Gaussian function as a weight. The closer *i and j* were, the higher the weight of the paired bases and the greater their effect.

**Figure 2:**
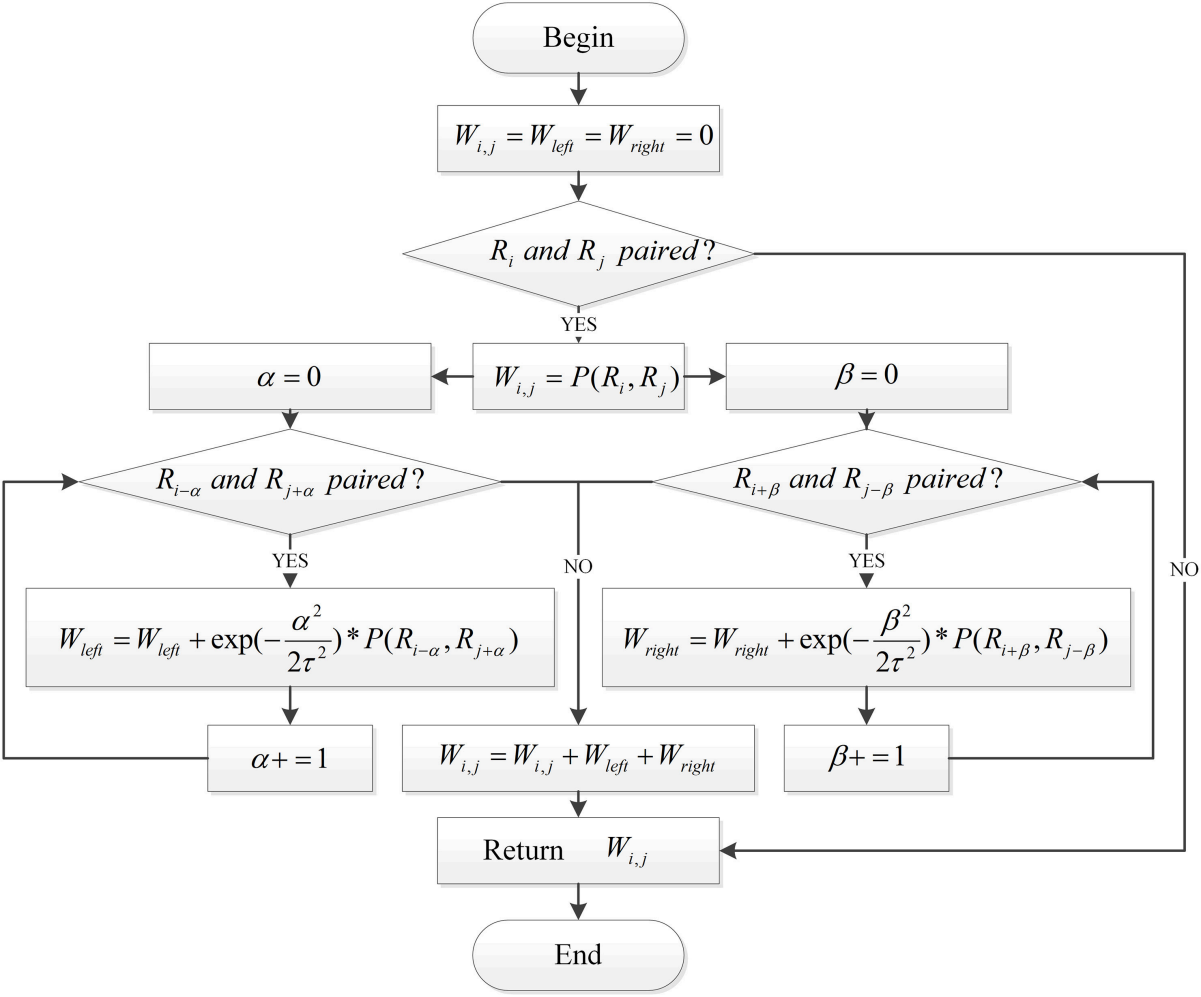
The process of RNA matrix representation based on RNA sequence pairing.

Combining these points of view, this paper introduces the following algorithm flow to calculate the specific values of each position of the coding matrix *W*_*i*×*i*_, as shown in Figure 2.

Base pairing according to RNA sequence coding matrix can be obtained by calculation. Through the analysis of the matrix, we can know that the position of the stem region in the real structure of the RNA is represented by a sub-diagonal line with a large intermediate value and a small value on both sides in the coding matrix. The advantage of the convolutional neural networks in deep learning methods is that they can effectively extract the regional features of the blocks in the matrix. Therefore, we used the convolutional neural networks instead of other machine learning models to predict the pairing of bases in RNA sequences.

### Convolutional Neural Network Predicts the Probabilities of RNA Sequence Base Pairing

Our goal is to predict the pairing of each base on an RNA sequence; so, we had to split the RNA sequence encoding matrix. The RNA representation method converts a sequence of length *n* into a matrix of size *n*× *n*. We use the sliding window method to divide the matrix into *n* matrices of size *d* × *n*. Where *d* is the size of the sliding window. Thus, the bases on each RNA sequence can be represented by a matrix of size *d* × *n*. The size of the sliding window, using the sliding window method, has a great influence on the experiment. If the sliding window is set too small, the extracted features will be incomplete. Too large a window setting will result in more redundant information in the matrix, which leads to a longer training model and may affect the accuracy of the final prediction model prediction. After analysis, the value of the sliding window should be related to the length of the stem region in the RNA. Therefore, we had to count the stem region information of the experimental object to determine the size of the sliding window.

The convolutional neural network requires that the data input into the model be of a uniform size, and the size of the RNA sequence corresponding to each RNA sequence is different due to the length of the RNA sequence. Therefore, during the experiment, we need to calculate the mean value of the RNA sequence length in the experimental data set and use that mean value to normalize the data. The sliding window method and normalization of the RNA coding matrix can convert the RNA sequence of length *n* into *n* matrices of the same size, which satisfies the requirements of the convolutional neural network for input data.

This article uses the dot bracket representation to represent the RNA secondary structure. The dot-bracket indicates that the RNA secondary structure is represented as a combination of sequences of “(“,”)” and “.”. Therefore, the output layer of the convolutional neural network designed in this paper is composed of three nodes, and the output of each base corresponds to the matrix corresponding to the probability of the three labels “(“, “)"and “.”.

### Maximum Probability Sum Algorithm Corrects Predictions

The deep learning method has a high accuracy rate for classification problems. However, RNA secondary structure prediction is not a simple classification problem. We can consider RNA secondary structure prediction as a combination of multiple classification problems under certain restrictions.

From the result of the previous step, we can obtain *P*_*left*_, *P*_*right*_, and *P*_*point*_. Which are the probabilities of the three labels “(“,”)” and “.” in the secondary structure of each base in the RNA sequence. However, if the label with the highest probability of prediction is used as the prediction result for each base, this combination does not guarantee that such a result will satisfy the definition of the secondary structure defined for RNA: It may appear that the number of left brackets is not equal to the number of right brackets or a prediction could be made in which the matched brackets cannot pair with the corresponding bases. So we need to modify the prediction results to meet the requirements of the definition of an RNA secondary structure.

Based on the probabilistic results obtained in the previous step of the convolutional neural network, the goal of this paper is to find a compatible bracketed sequence that represents the secondary structure of the RNA. To achieve this, the process requires:

1. Sequence Bracket Matching.
2. Matching brackets in the sequence that pair the bases in the corresponding positions (A-U,G-C,G-U).
3. To satisfy 1 and 2, the sum of the probabilities is maximized based on the output of each tag within its convolutional neural network.

To find a sequence that meets these requirements, this article enhances the Nussinov algorithm in the dynamic programming method. This requires changing the number of iteratively accumulated paired bases in the Nussinov algorithm to the sum of the cumulative probability of the iterative cumulative bases. Thus, a maximum probability sum algorithm was proposed. This algorithm makes use of the dynamic programming method. Through multiple iterations, the secondary structure of RNA that satisfies the requirements can be obtained. The specific iteration formula follows:

(2) $$\begin{array}{l} N(i,j)=\max\{\begin{array}{l} N(i+1,j)+p_{point}(R_{i})\\ N(i,j-1)+p_{point}(R_{j})\\ N(i+1,j-1)+\delta (R_{j},R_{j})\\ \underset{i<k<j}{\max}[N(i,k)+N(k+1,j)] \end{array}\\ \delta (R_{j},R_{j})=\{\begin{array}{l} p_{left}(R_{i})+p_{right}(R_{j})(R_{i\;}\;and\;R_{j}\;paired)\\ p_{point}(R_{i})+p_{point}(R_{j})(R_{i\;}\;and\;R_{j}\;not\;paired) \end{array} \end{array}$$

where *N (i, j)* is the maximum probability of the *i*-th base to the *j*-th base in the RNA sequence. *P*_*left*_*, P*_*right*_ , and *P*_*point*_represent the probability of the ith base of the RNA sequence being outputted by the convolutional neural network for three labels.

## Results

### Prediction of Secondary Structure of Single Family RNA by CDPfold

The data used in our experiment are derived from Turner and Mathews (2009). The data contained in the data set is shown in Table 1.

**Table 1:** Distribution of RNA types and their number in each dataset.

**RNA Type**	**Number**
5sRNA	1283
16sRNA	110
25sRNA	35
grp1RNA	98
grp2RNA	11
RNasePRNA	454
srpRNA	928
tmRNA	462
tRNA	557
telomeraseRNA	37

Among the various RNA families included in the dataset, we first selected the 5sRNA with the largest number and the most concentrated distribution without a pseudoknot. Sequence analysis of the 5sRNA dataset reveals that some identical or similar sequence data exists in RNA dataset. In order to avoid the effect of the experiments by the same or similar sequence data, it is necessary to preprocess the data in the dataset. That is, the 5sRNA data set is programmed to remove the same or similar sequences in the data. After the duplication removal operation, the number of 5s RNAs used in the experiment is 1,059. To train the model and accurately evaluate the entire model, we divide the number of removed 5sRNA datasets into a training set, consisting of a validation set and a test set. The ratio of RNAs in the training sets, validation sets and test set is 7:2:1. The experiment uses the training set to train the network model and determine the model parameters; then, the verification set is used to make the model selection. Therefore, in the final optimization and determination of the model, the final test set is used to measure the generalization ability of the whole prediction method.

**Figure 3:**
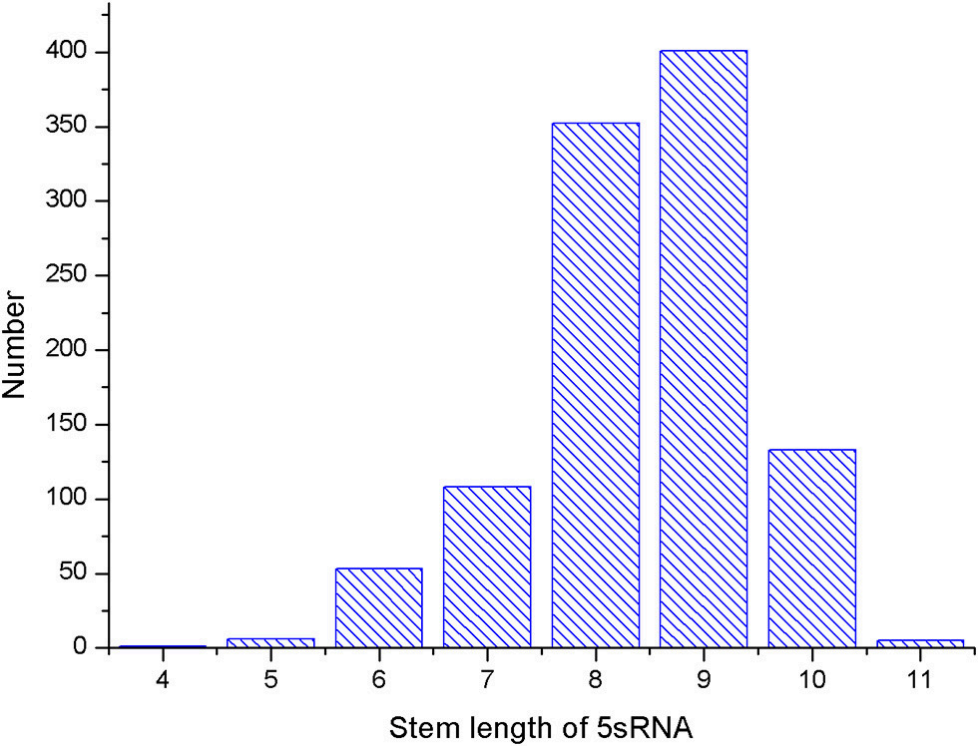
5sRNA maximum stem length statistics.

**Figure 4:**
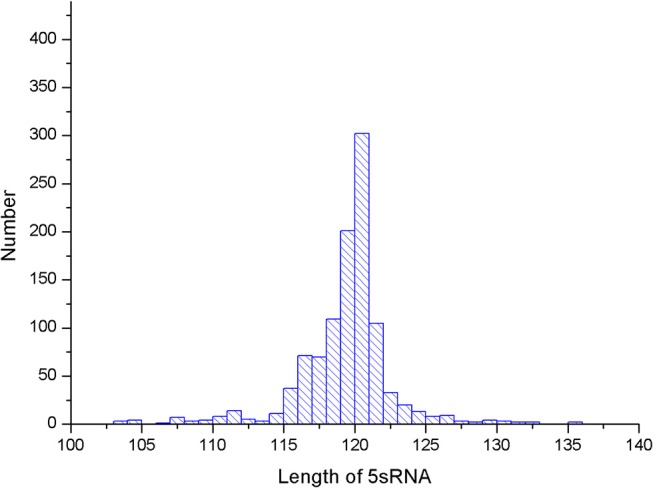
5sRNA length distribution.

Several parameters in the CDPfold can affect the results of the experiment, and the problematic parameters must be fixed before the experiment. The first problem parameter is the size of the sliding window. We calculated the length of the largest stem region of all RNAs in the 5sRNA data set used in the experiment. The results obtained are shown in the Figure 3. The length of the longest stem region in the 5sRNA dataset is used as the size of the sliding window method. We also calculated the average length of the 5sRNA sequence in the data set, as shown in Figure 4.

Figure 4 shows that the maximum stem length of the 5sRNA is 11 continuous base pair, and the average length of the sequence is 120 nt. Since the convolutional neural network has a good accuracy for the shifted and scaled images, this paper applies the idea of image scaling, which means that the matrix representation of the bases obtained through the sliding window can be uniformly scaled into a matrix size of 11 × 120.

**Figure 5:**
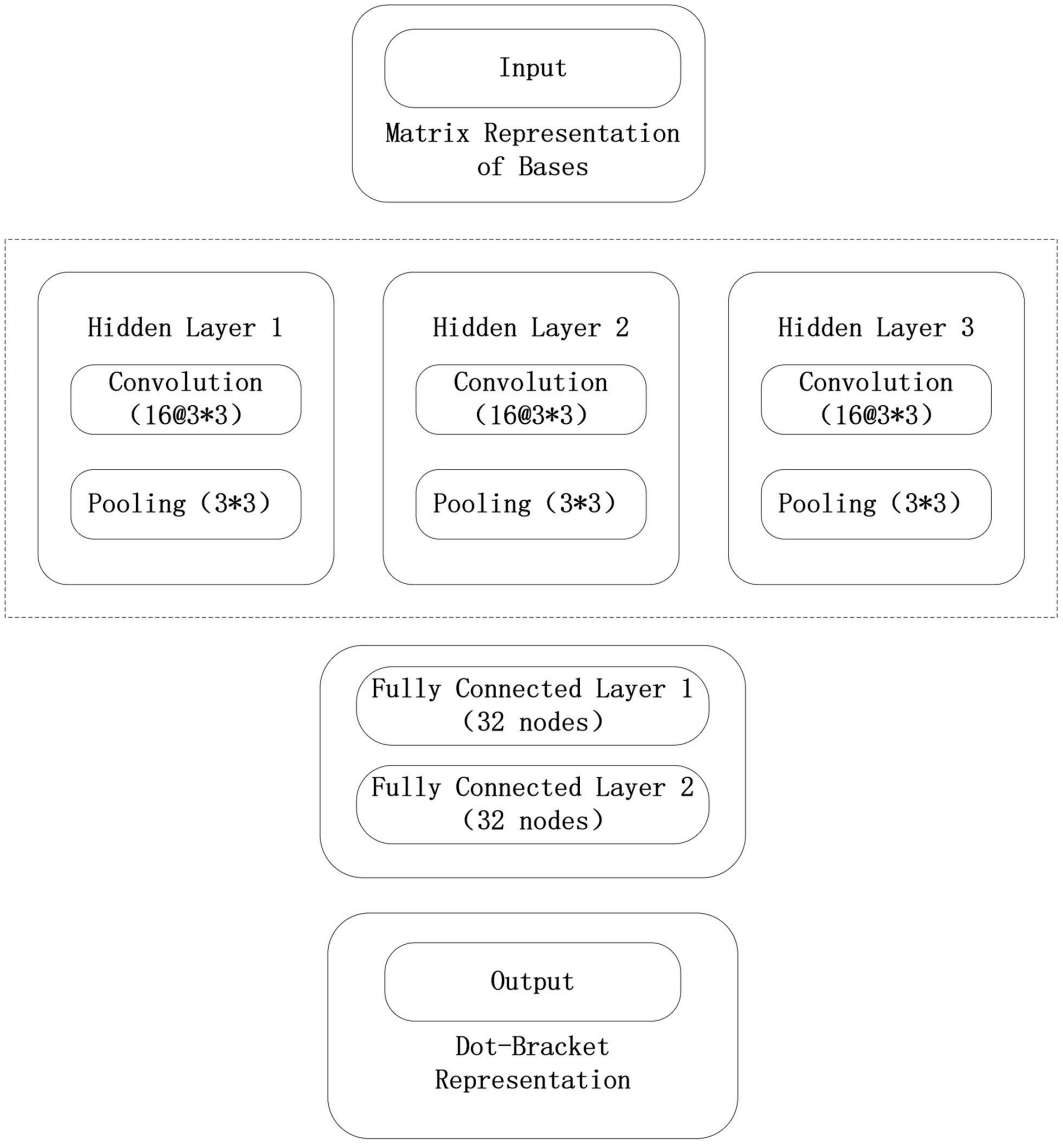
Convolutional neural network structure in the 5sRNA experiment.

The framework used in the convolutional neural network model constructed in this paper is Tensorflow. The convolutional neural network model consists of an input layer, three convolutional layers, three pooling layers, two fully connected layers, and a final output layer. In the test phase, the tf.nn.top_k() function of the output layer is removed to obtain the probability that each base will correspond to three tags. The convolutional neural network model used in this paper is shown in Figure 5.

The data input from the input layer of the convolutional neural network is represented by the sliding window algorithm and the normalized base matrix. The parameter optimization method using a batch random gradient descent uses 256 data for each iteration. The convolutional neural network consists of three convolutional layers, three pooling layers, and two fully connected layers, where each convolutional layer uses 16 3 × 3 convolution kernels, and each pool layer also uses 3 × 3 convolution kernels. The largest pool, and each full connection layer uses 32 nodes. The output layer of the model maps the data to the three labels of the point bracket representation, and the probability that the base belongs to three labels can be verified. The initialization method of each parameter of the model is the Xavier initialization method, and the error function of the output layer adopts the maximum entropy function. When the model parameters are trained, the model is iterated 400 times.

Through the sliding window method and normalization, a matrix representation corresponding to each base in the 5s RNA sequence in training set can be obtained, wherein each base has a corresponding structural label. Analysis of the data shows that because the number of unpaired bases in each 5s RNA sequence is slightly larger than the number of paired base pairs, this will result in an imbalance of the three types of data samples in the data set, so the data needs to be processed with unbalanced data. Since the amount of experimental data is sufficient, the upsampling data processing method will be adopted to balance the various sample data in the data set.

**Figure 6:**
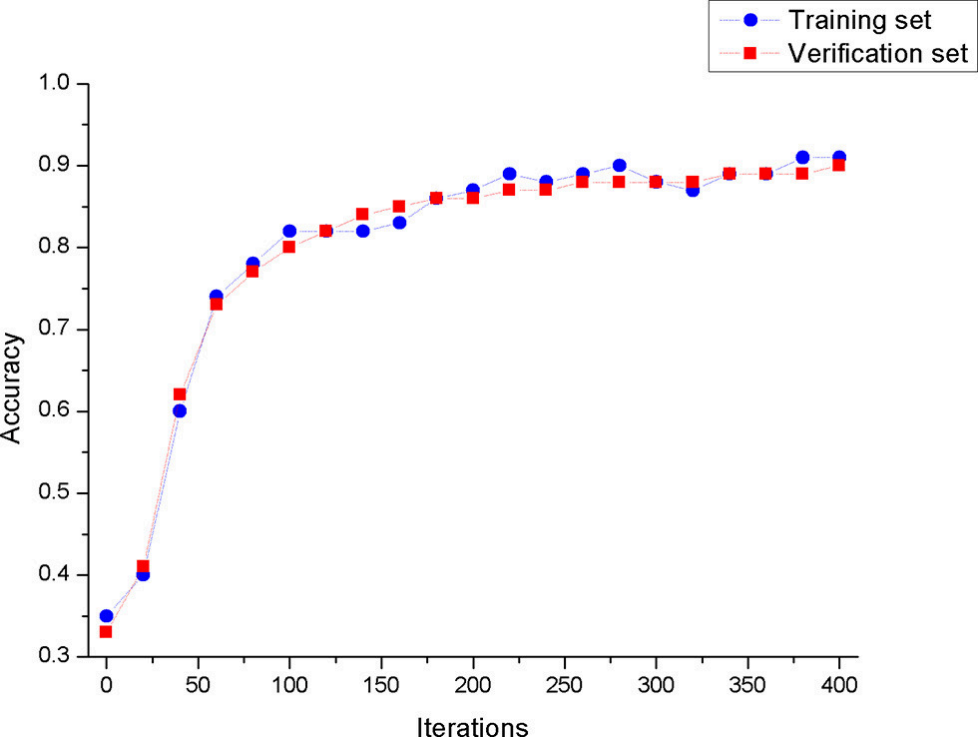
The accuracy of the model in the training set and the validation set.

The processed data is used to train the convolutional neural network model. The performance of the convolutional neural network model we built on the training set and test set is shown in Figure 6. From Figure 6, we can see that the model has a similar test accuracy on the training set and the test set, and the experimental results are not over-fitting. This figure also shows that the model has a similar test accuracy on the training set and the test set, and the experimental results are not over-fitting.

**Figure 7:**
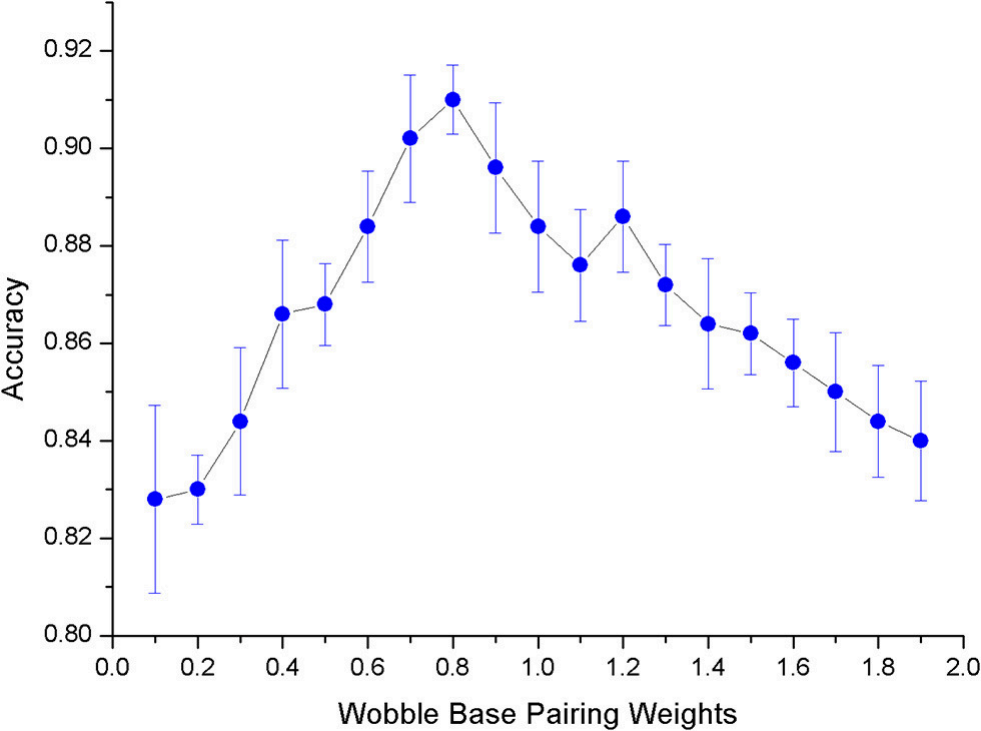
The ErrorBar of accuracy changes with wobble base pairing weights.

After determining the model used in the experiment, we need to select an appropriate value for the weight x of G-U pairing (Formula 1). The matching weight of the swing pair should not be too large or too small. Unfavorable weights will result in a decrease in prediction accuracy. In order to select the appropriate weights, we conducted a number of experiments. The results are shown in Figure 7. Experiments show that when the matching weight of G-U pairing is 0.8, the overall model's mean and variance of accuracy are optimal.

The test set data are input into the trained CDPfold, and the pairing probability of each base on each RNA obtained by the convolutional neural network is used as an intermediate result. These intermediate results are used in our probability and maximum correction algorithm. The optimal secondary structure that satisfies the definition of RNA secondary structure is obtained, and compared with the corresponding real structure, thereby validating our complete model design.

For the prediction of an RNA secondary structure obtained by the CDPfold, we used two indicators, sensitivity and specificity. Sensitivity refers to the predicted percentage of all base pairs in the real structure, corresponding to the recall-rate in machine learning. Specificity refers to the correct percentage of all predicted base pairs, corresponding to the precision-rate in machine learning. The RNA secondary structure prediction algorithm is difficult to achieve in general since it is always biased to one side. The *F*-score can be used to measure the precision and recall.

(3) $$\begin{array}{r} F\text{-}score=\frac{2\;\times \;Sensitivity\;\times \;Specificity}{Sensitivity\;+\;Specificity} \end{array}$$

Based on the above metrics, we obtained the predicted effects of the designed algorithm model on the 5sRNA dataset. We used the same data to perform experiments under other published algorithms. Table 2 compares the results of our experiments included in our new algorithm with the results obtained by other popular programs in current software. Table 2 shows the accuracy of our designed algorithm compared with other algorithms on the 5sRNA dataset. Obviously, the sensitivity and specificity of our designed algorithm are significantly higher than that found in other algorithms.

**Table 2:** Comparison of algorithms in 5sRna.

**Software**	**5sRNA**		
	**Sensitivity**	**Specificity**	***F*-score**
mfold	0.693	0.704	0.698
RNAfold	0.694	0.704	0.699
cofold	0.585	0.591	0.588
Sfold	0.703	0.733	0.718
CDPfold	0.932	0.916	0.924

### Prediction of Secondary Structure of Multiple Family RNAs by CDPfold

Based on the above studies, we used the 5sRNA dataset trained model to predict the secondary structure of tRNA. The results have a sensitivity of 0.2 and a specificity of 0.15.This is quite different from the 5sRNA dataset trained model effect on the 5sRNA data set. We analyzed this result and found the function of 5sRNA to quite different from that of tRNA. Models trained using the 5sRNA dataset only extract features that favor the classification of 5sRNA. The lack of these features in tRNA resulted in a greatly reduced prediction accuracy. Therefore, without determining the RNA function or family, it is not possible to directly predict those characteristics using the established model. Thus, the entire sample data must be obtained to build a general model.

First, we analyzed all the data in the dataset and found pseudoknots in some RNA structure data. Since the pseudoknot belongs to RNA tertiary structure category, all data with pseudoknots were deleted in the pre-processing operation. Table 3 shows the number of RNAs after the pseudoknot deletion.

**Table 3:** The number of RNAs in each data set before and after the pseudo-knot was removed.

**RNA Type**	**Before**	**After**
5sRNA	1283	1283
16sRNA	110	50
25sRNA	35	20
grp1RNA	98	0
grp2RNA	11	11
RNasePRNA	454	37
srpRNA	928	928
tmRNA	462	3
tRNA	557	557
telomeraseRNA	37	0

**Figure 8:**
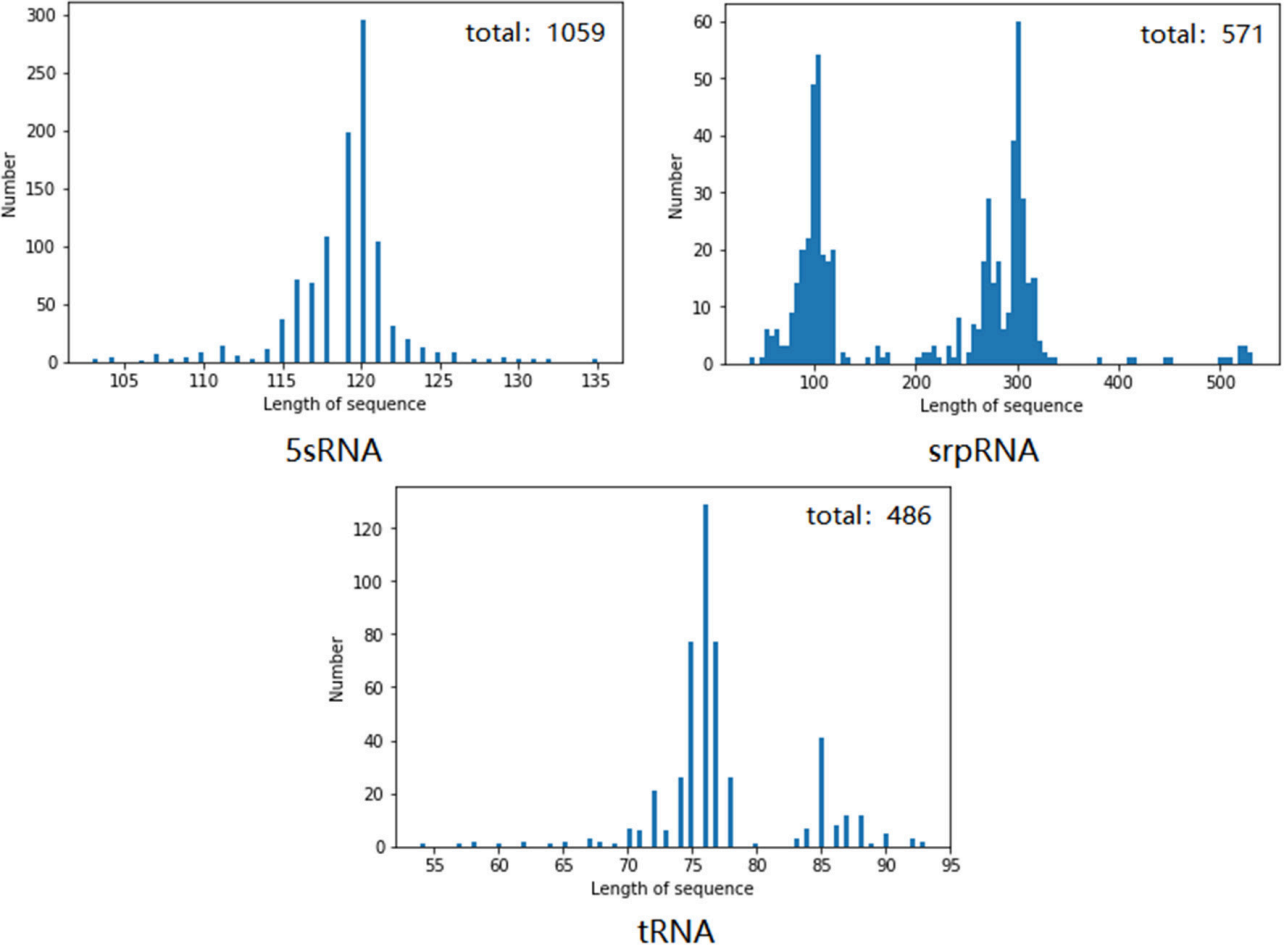
Number and length distribution of the RNA data of each family after redundancy.

We chose the 5sRNA, srpRNA, and tRNA using a number >100 after the pseudoknot, and first performed removal of redundant operations on these three types of RNA to delete the same or similar sequence data in the RNA data set. Figure 8 shows the RNAs of each family and the data distribution after the removal of identical or similar sequence data.

**Figure 9:**
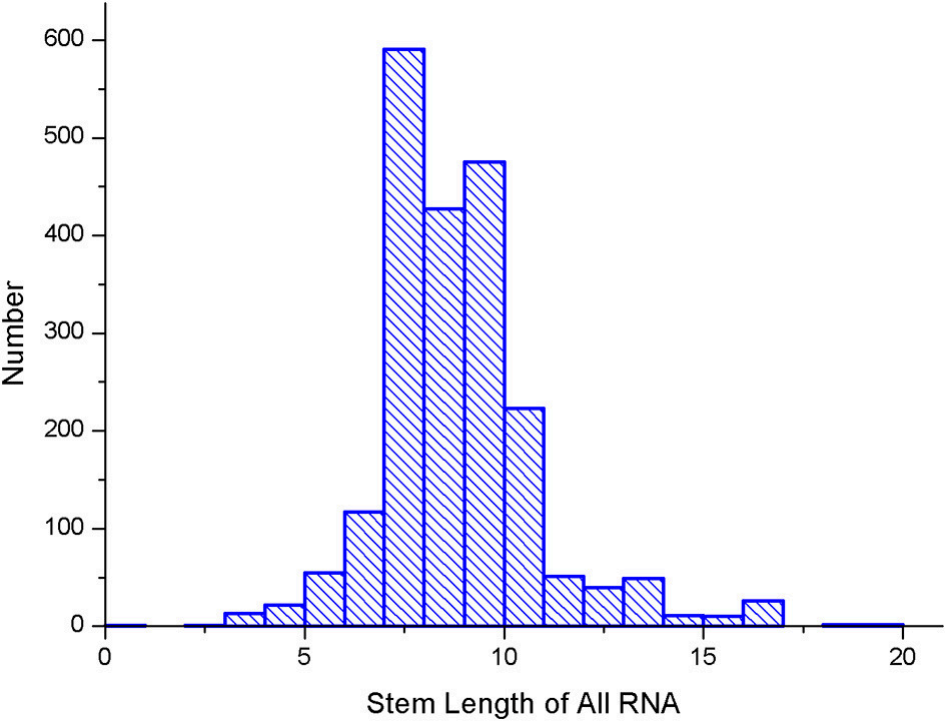
Stem length statistics in the data set.

**Figure 10:**
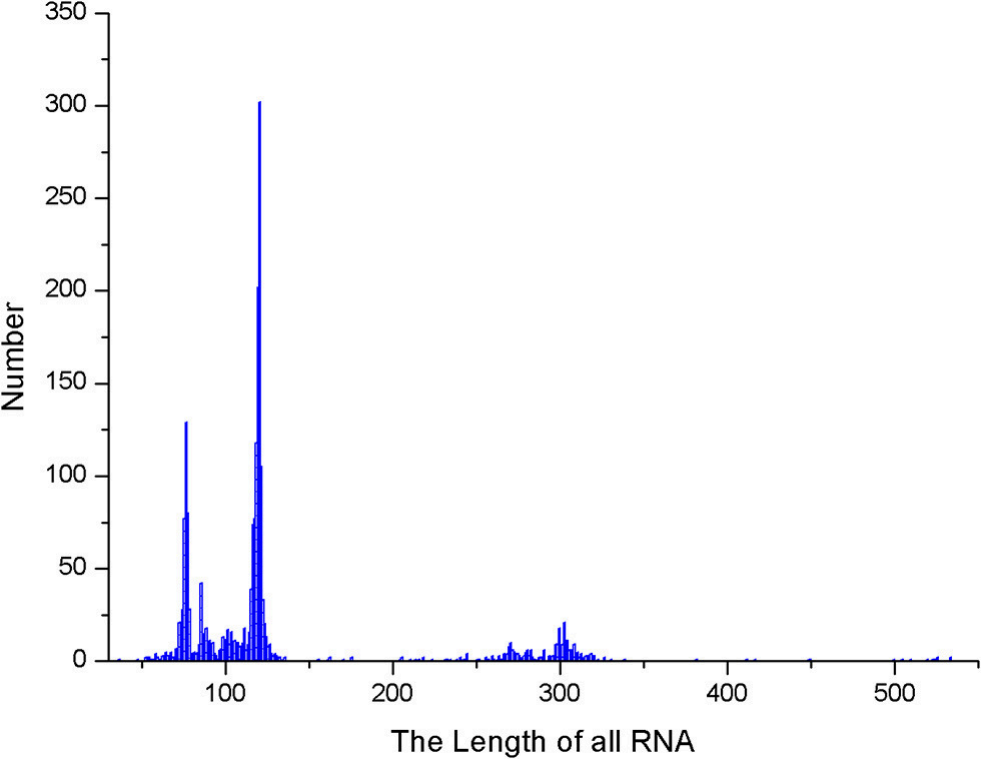
RNA sequence length distribution.

We also calculated the maximum stem length of each RNA sequence in the data set and the average length of the RNA sequence, which are shown in Figures 9, 10.

**Figure 11:**
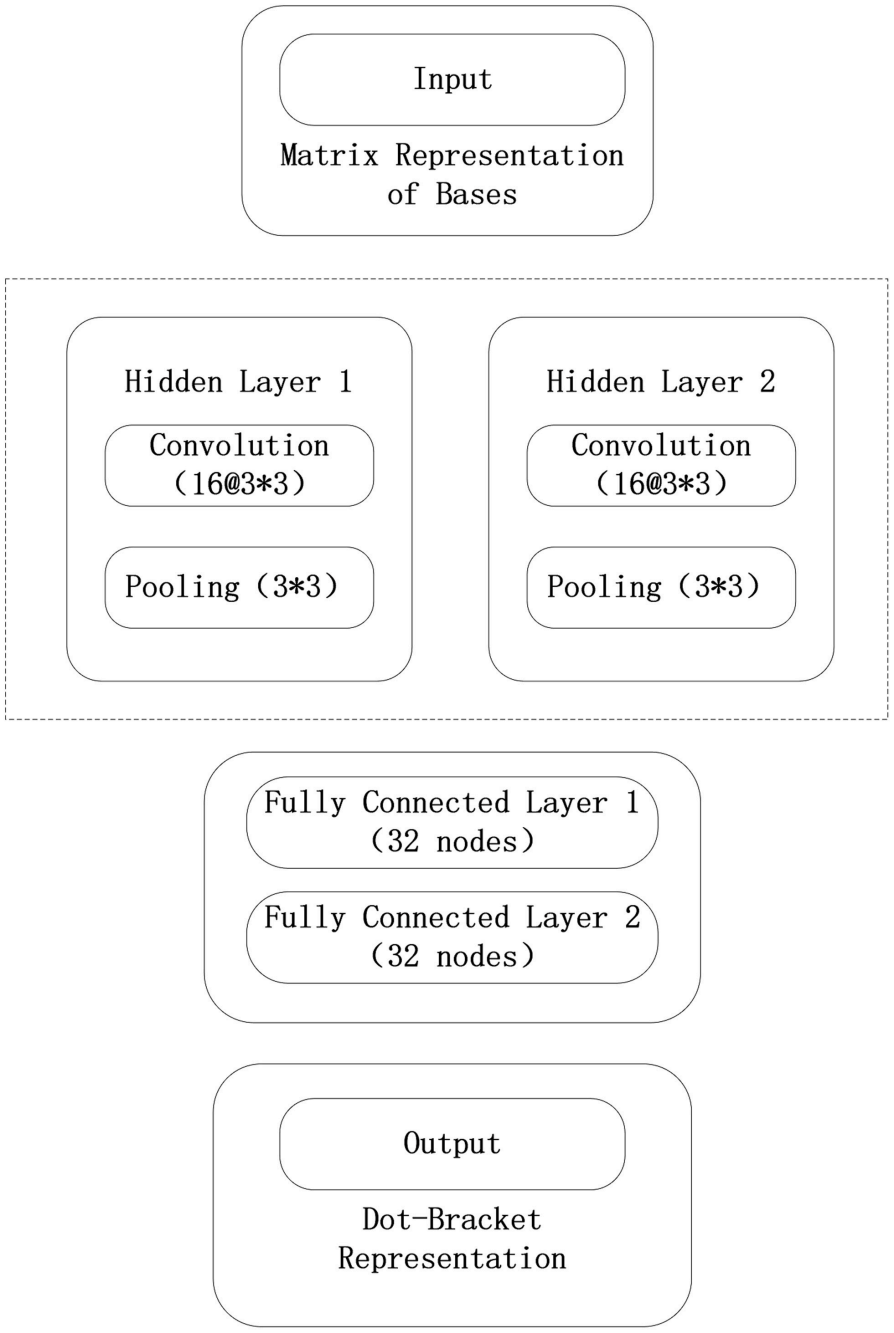
Convolutional neural network model in general model.

As it can be seen from Figures 9, 10, the maximum length of the stem region is 19 continuous base pair, and the average length of the sequence is 128 nt. Thus, the RNA matrix representation will be represented as a matrix of size 19 × 128 after normalization and sliding window operations. The experiment divides the data into training sets, validation sets and test sets, in which the ratio of various types of RNA remained at 7:2:1. In the general model, due to the significant increase in the number of data types and RNA, the experiment will fine-tune the convolutional neural network of the original 5sRNA prediction model: To extract the more generalized hidden features of various RNAs, the number of convolutional and pooling layers is reduced from three to two. Other configuration parameters have not changed. During the training process, the batch data size for each iteration is increased from 256 to 512, and the number of iterations is increased to 2,000. In Figure 11, the convolutional neural network model used in the experiment is as follows.

After our convolutional neural network model is trained, the test set data is input into the trained general model to obtain the pairing probability of each base on each RNA, and the maximum probability and base correction algorithm are used for pairing probability and RNA sequence. In this manner, the optimal secondary structure of the RNA sequence is obtained.

Using the F-score, we can get the predicted effect of the designed generic model on the three types of RNA datasets. We use the same test data to perform experiments under other published algorithms. The comparison results are shown in Table 4.

**Table 4:** Comparison of three types of RNA based on their prediction accuracy.

**Software**	**5sRNA**	**tRNA**	**srpRNA**
Mfold	0.698	0.631	0.566
RNAfold	0.699	0.632	0.577
CDPfold	0.911	0.905	0.823

## Discussion

This paper proposes a CDPfold prediction method based on a convolutional neural network for RNA secondary structure. This method uses the convolutional neural network to extract the hidden features of RNA sequence data and applies it to the field of structural prediction. The results are corrected using a dynamic programming-based correction algorithm to obtain an optimal RNA secondary structure. Experimentally, our method has had good performance in predicting the accuracy of a RNA secondary structure.

Although CDPfold has achieved good results in RNA secondary structure prediction, some problems encountered during the experiment process are summarized below, and suggestions for solving the problem follow.

First, the reason why RNA can form stem regions depends on the hydrogen bonds formed by the complementary pairing of bases. The secondary structure of DNA mainly exists in the form of a double helix. Due to the limitation of double helix structure, base pairing in DNA can only be composed of pyrimidines and hydrazine pairs. Therefore, DNA molecules only have two pairing modes: A-T and G-C.RNA molecules are different. RNA molecules mainly exist in single-stranded forms. Their double-stranded regions are composed of different regions of the same chain. They do not have a long structural regular double-helix structure. Therefore, in addition to standard A-U and G-C base pairs, there are also G-U swing pairs. The hydrogen bonds formed by the rocking pair are unstable, and not all G and U elements can form paired base pairs. The current handling of G-U swing pairings will be fixed, either as pairable or as unpairable. In this paper, a smaller number is selected as the pairing weight on the G-U swing pair, but the G-U pairing problem is not well-explained. We believe that it is necessary to dynamically determine whether G and U can form paired base pairs according to different states during the process of RNA folding, but this dynamic method is extremely difficult, and there is no research to propose a corresponding solution.

The results predicted by the CDPfold method proposed in this paper still need to be further corrected in the results predicted by the convolutional neural network. This is because all machine learning algorithms have generalization errors, and the convolutional nerves are caused by the existence of generalization errors. The results obtained by the network did not form a satisfactory RNA secondary structure. A similar situation has emerged in other studies that use machine learning algorithms to solve RNA secondary structure predictions. There are two main solutions to obtaining a satisfactory RNA secondary structure for more accurate predictions. One is to directly optimize the results of the machine learning model. This paper adopts this approach. The second is to use the results as conditional constraints, and use these constraints to optimize other algorithms. In essence, both approaches are an optimization process for intermediate results. In this problem, it may be an effective solution to generate an anti-network model. The generator that generates the anti-network is used to generate the RNA secondary structure, and the discriminator is used to determine whether the results satisfy the definition of the RNA secondary structure. The optimal RNA secondary structure is obtained by the confrontation between the generator and the discriminator. The difficulty of this method is how to design a good training method. Otherwise, the output may be unsatisfactory due to the freedom of generating the model.

In the selection method of an optimization algorithm, the authors of this paper used group intelligence optimization algorithms such as genetic algorithm. These intelligent algorithms can solve complex non-linear problems by simulating biological evolution. In this paper, the probabilistic results provided by the convolutional neural network are used as the probability of selection, mutation, hybridization, etc. in the genetic algorithm, and the number of mismatches in the simulated RNA structural species is used as the optimization target. Although this method can also obtain the RNA secondary structure that meets the requirements, the randomness of each link in the group intelligent optimization algorithm and the discreteness of the data prevent the algorithm from having a fixed number of optimization iterations. In addition, since the goal is to find the secondary structure of the RNA that does not mismatch, and the number of such results is large, the result of each optimization is uncertain, so the group intelligence algorithm cannot be used as the optimization algorithm of this paper. Therefore, the dynamic programming algorithm was chosen as the optimization algorithm, and the probability and maximum correction method are proposed based on the Nussinov algorithm.

In the current prediction of the RNA secondary structure, the prediction of pseudoknots is still a difficult point. In this study, it was found that 5sRNA, srpRNA, and tRNA are free of pseudoknots, while most of RNasePRNA and tmRNA have pseudoknots. In these RNAs containing pseudoknots, the number of pseudoknots in each RNA is relatively small, but their existence cannot be ignored. Not only pseudoknots plays an important role in the function of the RNA, but also the prediction of the pseudoknot effect is wrong, it will cause a mistake in the normal stem area. The RNA structure representation method used in this paper uses the dot bracket representation. However, the dot bracket representation does not reflect the false knots present in the RNA structure. Therefore, the data containing the pseudoknots are deleted in the experiment. If a secondary structure representation of RNA can be found that can represent a pseudoknot, the CDPfold proposed in this paper can be modified accordingly to predict the secondary structure of the RNA with a pseudoknot.

The experimental data used in this paper focuses on 5sRNA, srpRNA, and tRNA. The length of these three types of RNA sequences is mostly between 50 and 350 nt. In this part of the length range, the effect of CDPfold is due to the existing RNA prediction software. The prediction of the secondary structure of longer RNA sequences is not reflected. This is because the current experimental methods are not perfect enough. The secondary structure data of long-sequence RNAs measured by experiments are not enough. The data set was provided by Turner and Mathews (2009) used in the experiment. Less than 200 RNA sequences longer than 1,000 nt were used, which is <10% of the entire data set. The most important factor affecting the predictive effect of deep learning is the amount of data, so we did not study longer sequences. However, with the continuous improvement of experimental techniques, the number of long sequence structures measured by experiments continues to increase. On this basis, models based on deep learning have an advantage.

The last point is the instability of the RNA structure. The structure of the RNA molecule is highly susceptible to environmental factors. Studies have shown that RNA molecules can damage their natural structures when they are exposed to an *in vitro* environment, leading to structural damage; thus, *in vivo* structural prediction experiments are not perfect, which means the current RNA secondary structure is not necessarily a real structure.

In addition, unlike proteins, which function differently, not all RNA molecules can function in the body; furthermore, RNA that encodes proteins accounts for only 2% of the total RNA. Thus, RNA structures that do not have an actual function may not be as fixed as functional RNA structures. These problems all have an impact on the prediction of RNA secondary structure prediction.

In general, the CDP-Fold algorithm based on the convolutional neural network for RNA secondary structure prediction achieved good results in data sets without pseudo-knots. Many difficulties remain in the research of RNA secondary structure prediction, and many parts still need to be improved. Our research provides new ideas for the study of the RNA secondary structure and serves as a very good source of structural prediction problems and solutions for other researchers.

## Data Availability

Publicly available datasets were analyzed in this study. This data can be found here: https://github.com/zhangch994/CDPfold.

## Author Contributions

YL, HZ, and ZL conceived and directed the project. CZ, CL, and XW obtained the raw data and interpreted the data. HZ, CZ, and ZL conducted the data analysis and interpreted the results. HZ, CZ, XW, and BZ helped to design the study and reviewed the data. YL, HZ, CZ, and BZ wrote and edited the manuscript. All the authors helped with the draft and reviewed the manuscript before approving for publication.

### Conflict of Interest Statement

The authors declare that the research was conducted in the absence of any commercial or financial relationships that could be construed as a potential conflict of interest.
